# Supplementary material for: Topological analysis reveals state transitions in human gut and marine bacterial communities
Source: NPJ Biofilms Microbiomes. 2020 Oct 14;6:41. doi: 10.1038/s41522-020-00145-9 (PMC7560872; doi:10.1038/s41522-020-00145-9)
Supplement: Supplementary file 1 — Supplementary Information [file 41522_2020_145_MOESM1_ESM.pdf]

Supporting information: Topological analysis reveals state transitions in human gut and marine bacterial communities

William K. Chang, Dave VanInsberghe, Libusha Kelly

July 21, 2020

1 Supplementary Data Files

- `cholera-state-compositions.txt`: taxonomic table showing the mean composition of each state for the cholera data set
- `david-state-compositions.txt`: taxonomic table showing the mean composition of each state for the two adult human gut microbiomes data set
- `prochlorococcus-state-compositions.txt`: taxonomic table showing the mean composition of each state for the *Prochlorococcus* data set

2 Supplementary Methods

2.1 PCA and hierarchical clustering

PCA plots were generated using the prcomp function in the R package vegan v2.5-4 [1] and visualized using the R package ggplot2 v3.2.1 [2]. To perform heirarchical clustering analysis, bray-curtis dissimilarity values between samples within each dataset were calculated using the function vegdist in the R package vegan v2.5-4, and clustered using the R function hclust.

Supplementary References

[1] Jari Oksanen et al. *vegan: Community Ecology Package*. Version 2.5-6. Sept. 1, 2019. URL: https://CRAN.R-project.org/package=vegan (visited on 03/02/2020).

[2] Hadley Wickham. *ggplot2: Elegant Graphics for Data Analysis*. Google-Books-ID: XgFkDAAAQBAJ. New York, NY: Springer-Verlag, June 8, 2016. 266 pp. ISBN: 978-3-319-24277-4.

3 Supplementary Tables

Data set	Sampling frequency	Duration	Notes
Cholera	irregular: multiple times/day during diarrhea, daily for first week after discharge, weekly over next 3 weeks of recovery, monthly over next 2 months	33-76 hours diarrhea, 86-88 days recovery	7 patients
Two adult human gut microbiomes	$\sim$ daily	252 days (subject B)-364 days (subject A)	2 subjects
Prochloroccocus	$\sim$ monthly	$\sim$ 5 years	2 sites, 12 depth fractions each

Table 1: Description of sampling frequencies and experiment duration for all data sets analyzed.

4 Supplementary Figures

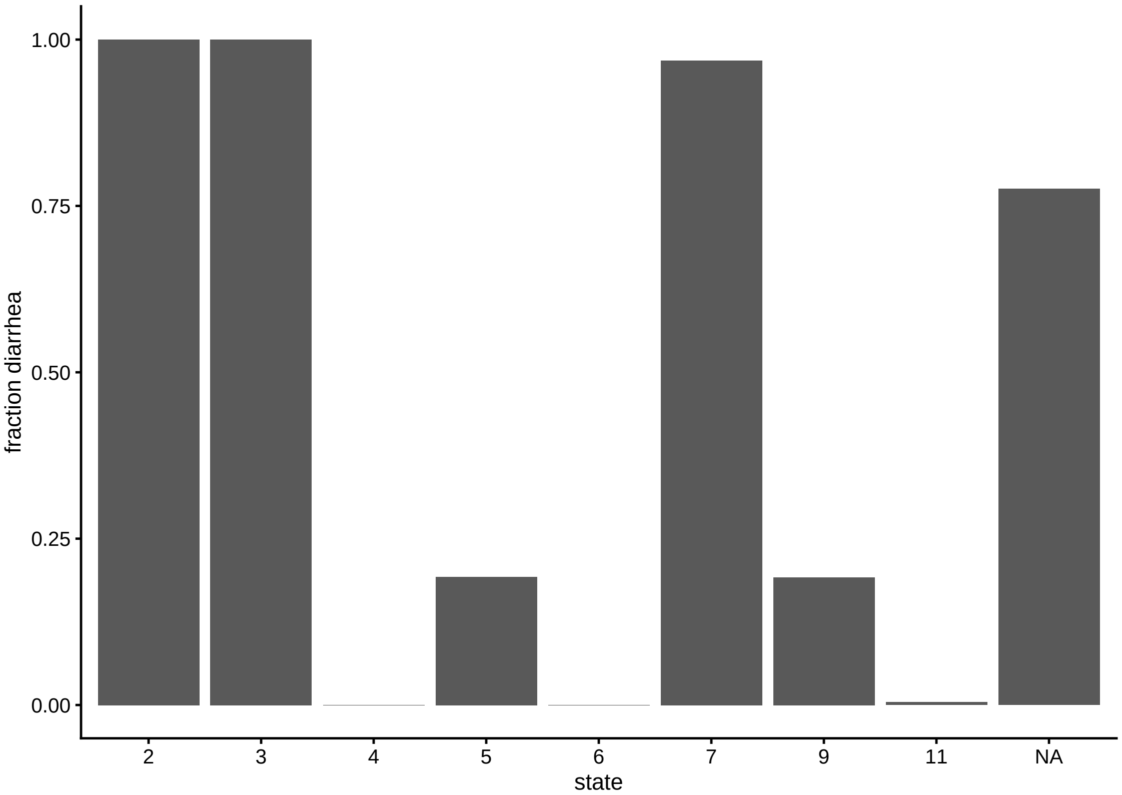

Figure 1: Mean fraction diarrhea per vertex for each state for the cholera data set.

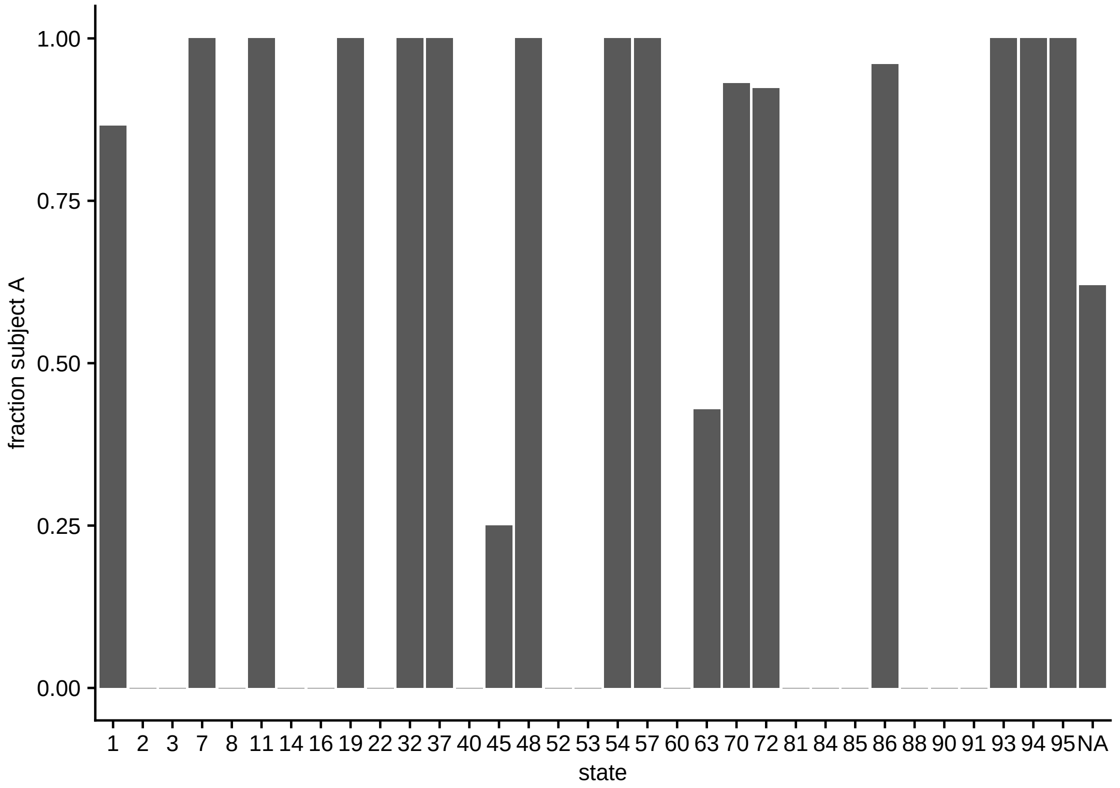

Figure 2: Mean fraction subject A per vertex for each state for the two adult gut microbiomes data set.

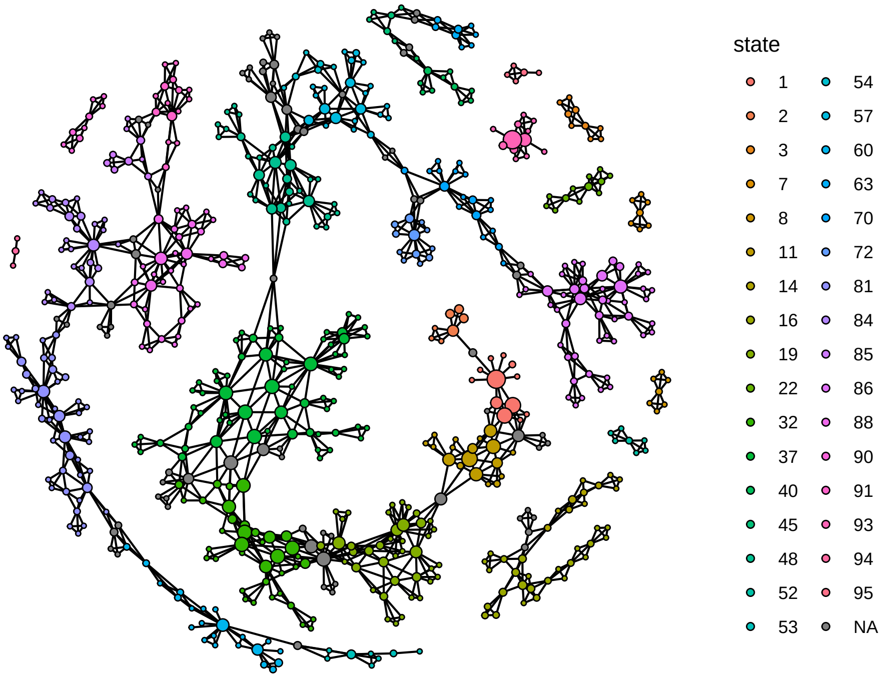

Figure 3: Metastable states for the data set of two healthy adult male gut microbiomes.

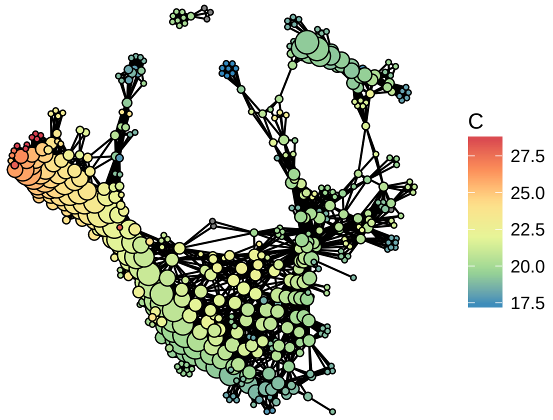

Figure 4: Mapper representation of the *Prochlorococcus* phase space colored by mean temperature per vertex. The phase space is spanned by a continuous temperature gradient.

.

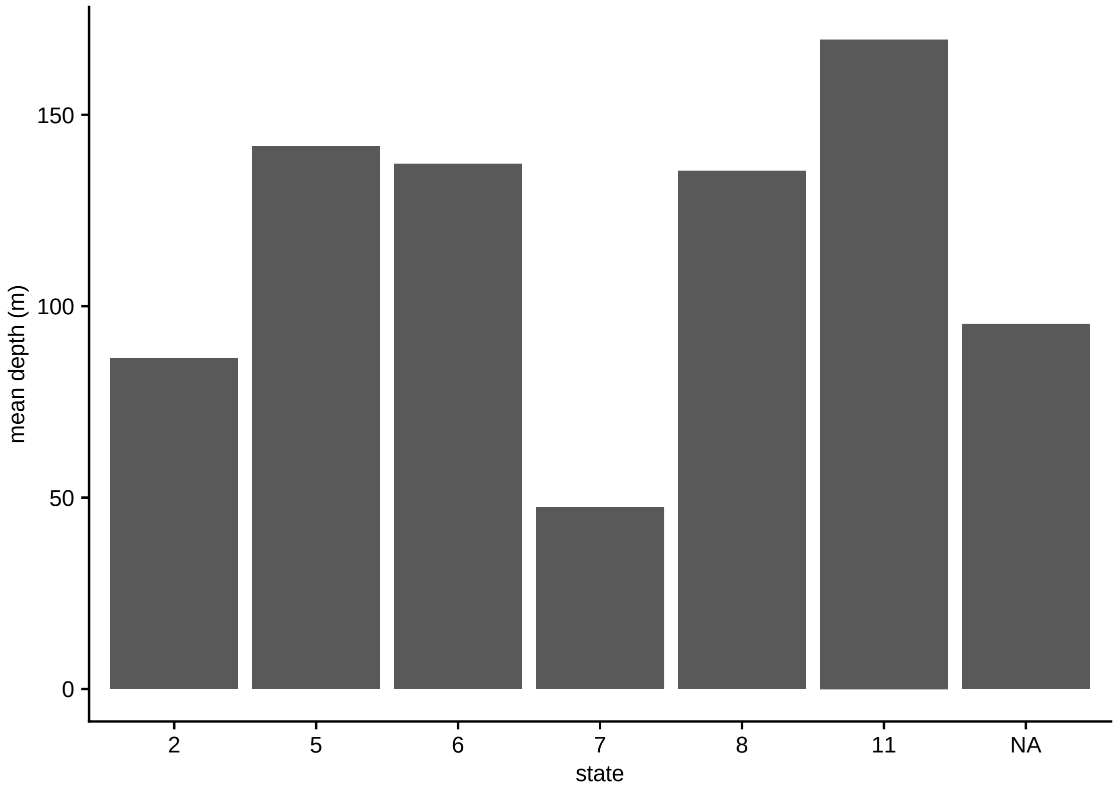

Figure 5: Mean depth per vertex for each state for the *Prochlorococcus* data set.

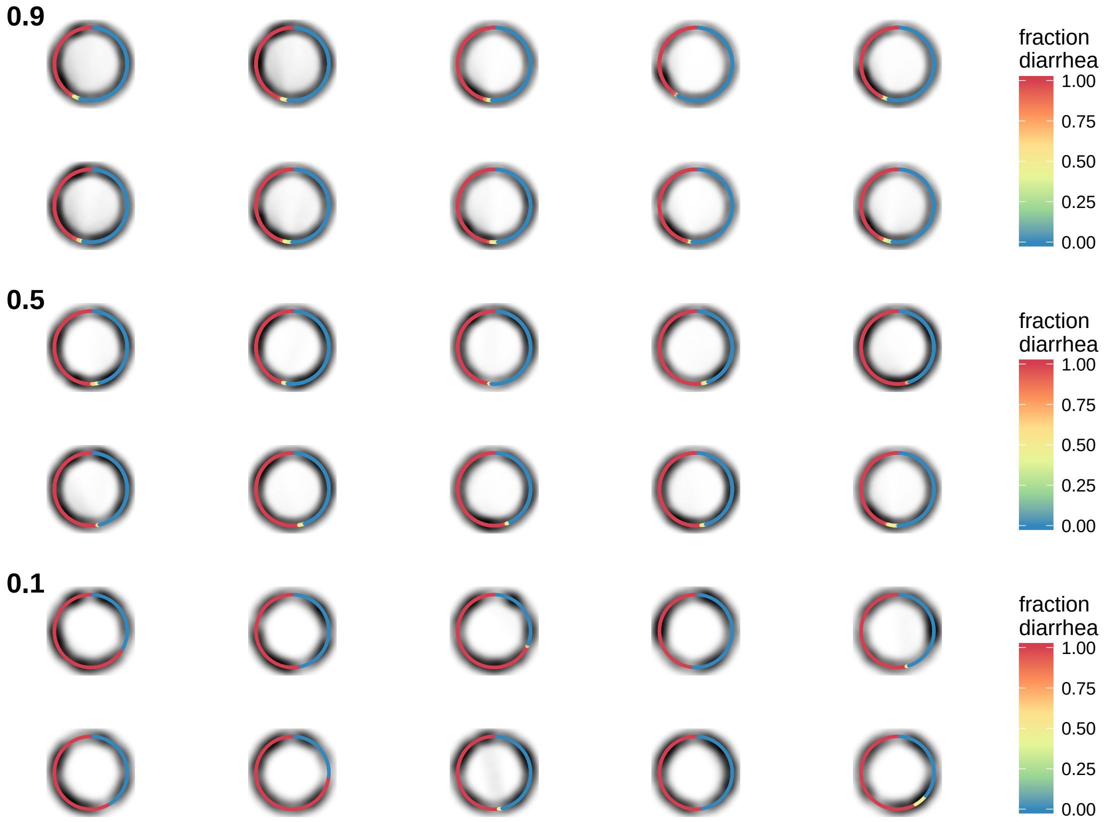

Figure 6: Mapper representations of 10 random subsets of the cholera data constituting 0.9 (top), 0.5 (middle), and 0.1 (bottom) of total data points, each. Nodes of Mapper graph laid out linearly and ordered by fraction of samples corresponding to phenotype, from 0% diarrhea (blue) to 100% (red). Gradient represents edge density. The majority of edges link nodes of similar phenotype, with few connections between the diarrhea and recovery regions of phase space.

.

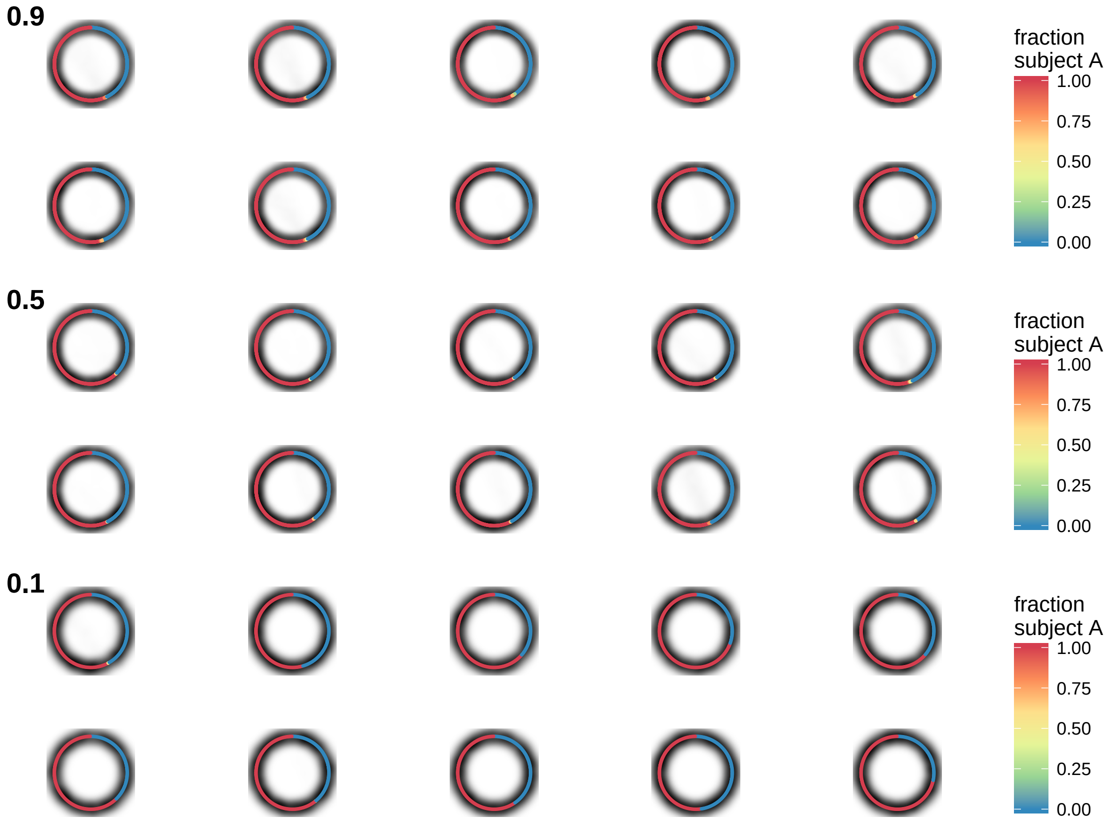

Figure 7: Mapper representations of 10 random subsets of the healthy human adult data constituting 0.9 (top), 0.5 (middle), and 0.1 (bottom) of total data points, each. Nodes of Mapper graph laid out linearly and ordered by fraction of samples corresponding to subject, from 0% subject A (blue) to 100% (red). Gradient represents edge density. The majority of edges link nodes representing the same subject.

.

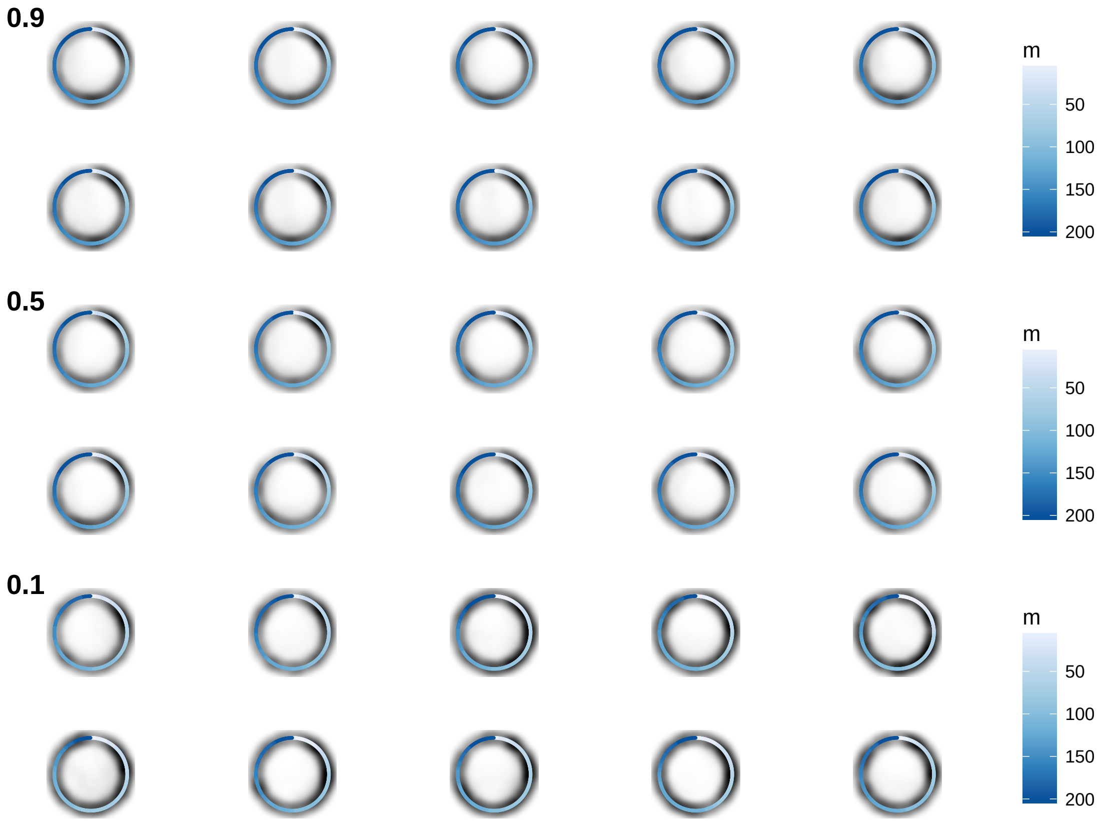

Figure 8: Mapper representations of 10 random subsets of the *Prochlorococcus* data constituting 0.9 (top), 0.5 (middle), and 0.1 (bottom) of total data points, each. Nodes of Mapper graph laid out linearly and ordered by mean depth, from shallow (white) to deep (blue). Gradient represents edge density. The majority of edges link nodes with similar depths,

.

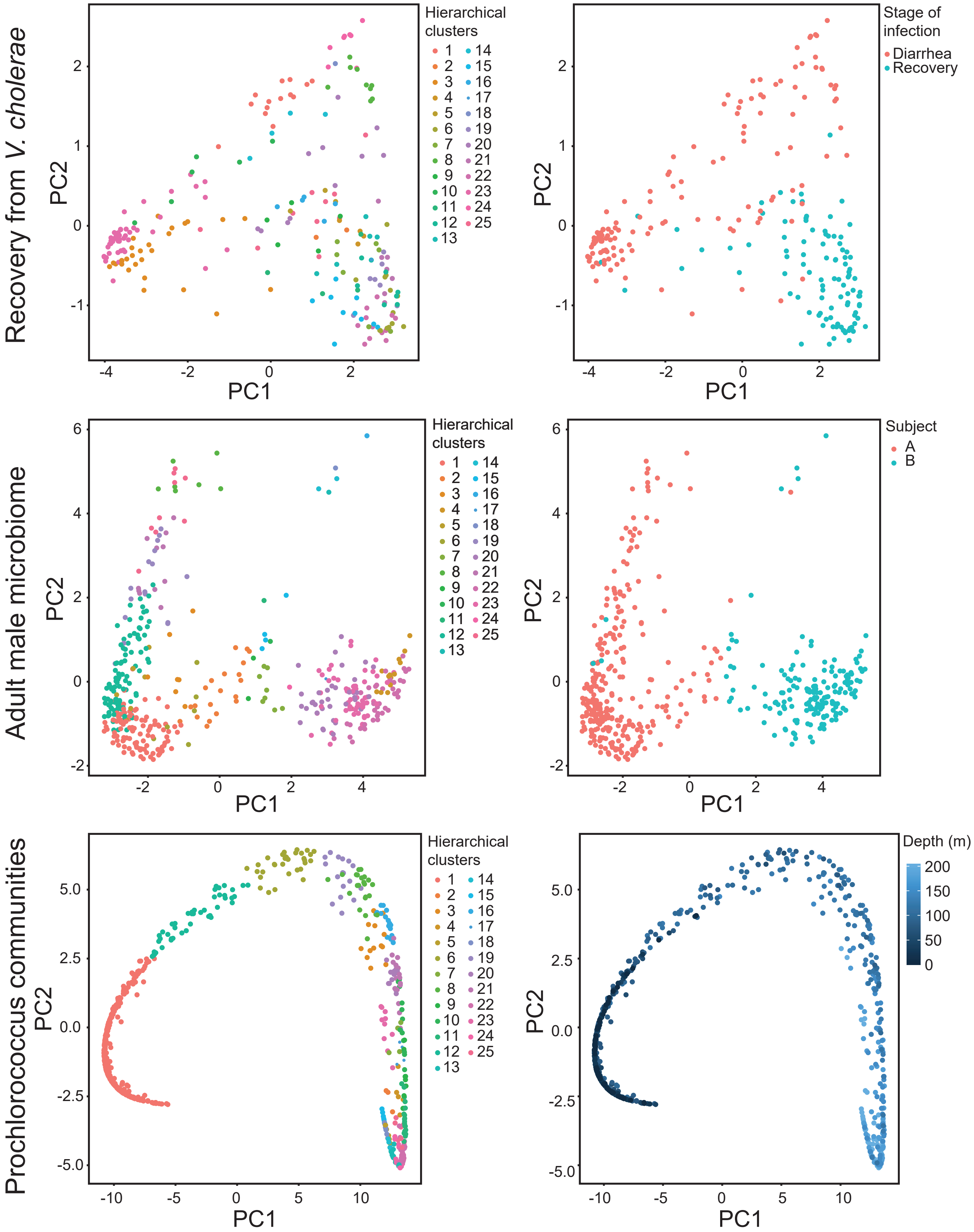

Figure 9: Principal component analysis of time series microbial communities. Samples are colored according to hierarchical clusters (left) and attributes of the person or depth they were taken from.
